# Ameliorative Effects of Liquiritin Carbomer Gel on Dinitrofluorobenzene-Induced Atopic Dermatitis in Mice

**DOI:** 10.3390/gels12040328

**Published:** 2026-04-14

**Authors:** Yun Zhang, Qiqing Tan, Sijia Li, Xiangdi Hu, Aoxiang Luo, Ming Li

**Affiliations:** 1 School of Nursing, Guangdong Pharmaceutical University, Guangzhou 510310, China; zhangyun@gdpu.edu.cn (Y.Z.); tqq20000609@163.com (Q.T.); lisijia22@163.com (S.L.); 18927779344@163.com (X.H.); 2 School of Basic Medical Sciences, Guangdong Pharmaceutical University, Guangzhou 510006, China

**Keywords:** liquiritin, atopic dermatitis, ameliorative effects, inflammation, skin barrier

## Abstract

Atopic dermatitis (AD) is a chronic inflammatory skin disease characterized by dryness and itching. Steroids are the most common therapeutic agents, may induce skin atrophy, and damage the skin barrier. Therefore, we need to find a safer alternative option. Liquiritin (LQ), a flavonoid compound extracted from licorice rhizomes, possesses anticancer, anti-inflammatory, and antioxidant effects. This study aimed to investigate the therapeutic effects of LQ on AD, focusing on its potential skin barrier-protective and anti-inflammatory mechanisms. In this research, we prepared liquiritin carbomer gel (LQ-CG) and assessed its treatment effects on mice with AD triggered by 2,4-dinitrofluorobenzene (DNFB). It effectively attenuated AD progression by ameliorating skin lesions, decreasing epidermal thickness and mast cell infiltration, downregulating inflammatory cytokine levels, and restoring the expression of claudin-1, loricrin, and occludin. It also inhibited the release of TNF-α, IL-1β, and IL-6 in lipopolysaccharide (LPS)-stimulated RAW264.7 cells, and showed no significant toxicity to major organs in mice. In summary, our findings demonstrate that LQ-CG can effectively alleviate atopic symptoms by repairing the skin barrier and inhibiting inflammatory responses without causing significant changes in organ indices

## 1. Introduction

Known as atopic eczema, atopic dermatitis (AD) is a persistent skin disorder that features immune system irregularities and weakened skin barrier, leading to inflammation. Clinically, it manifests as erythema, dryness, edema, erosion/desquamation, exudation, crusting, and lichenification, accompanied by an increased susceptibility to bacterial and fungal infections [1]. Driven by environmental changes and escalating psychosocial stress, the global prevalence of AD has progressively increased, with current epidemiological data indicating rates of up to 25% in children and over 10% in adults [2]. The severe discomfort and diverse skin lesions of AD often lead to psychological issues such as low self-esteem, anxiety, and depression, which significantly impact daily life and academic performance [3]. A study by Liu involving 273 patients with AD revealed that 24.5% and 19.8% experienced symptoms of anxiety and depression, respectively [4]. Furthermore, these symptoms may progress to other allergic conditions, including asthma, food allergies, allergic conjunctivitis, and allergic rhinitis [5]. AD is currently the most burdensome dermatological disease globally and ranks as the 24th leading cause of non-fatal health loss in China [6]. AD has serious consequences and imposes profound psychological and social burdens on patients; therefore, exploring safe and effective therapeutic strategies has become an important and urgent challenge in dermatology.

The intact structure and proper function of the epidermal barrier is essential for maintaining skin stability and serves as the primary physicochemical and immunological shield against environmental insults. A hallmark of AD is compromised skin barrier. The stratum corneum, the outermost skin layer, constitutes the principal permeability barrier. Its formation relies on the terminal differentiation of epidermal keratinocytes into protein-rich corneocytes, which are subsequently embedded within a lamellar, lipid-rich extracellular matrix [7,8]. This organization conforms to the “brick and mortar” model, where corneocytes represent the bricks, surrounded by a matrix mainly comprising ceramides, cholesterol, and free fatty acids [9]. Underlying the corneocyte plasma membrane, the cornified envelope incorporates structural proteins such as loricrin to confer mechanical resilience [10]. Within the stratum granulosum, keratinocytes are interconnected by tight junctions (TJs), transmembrane protein complexes that create a paracellular seal. Among these, claudin-1 is critical for determining barrier selectivity and integrity, while occludin contributes to its dynamic stability and regulatory functions [11]. Any impairment in the synthesis, assembly, or maintenance of these constituent elements culminates in a barrier defect.

AD not only arises from skin barrier dysfunction but also dysregulated immune responses [12]. Central to this dysregulation is the T-helper 2 (Th2) immune axis, which drives inflammation and further compromises barrier integrity, creating a self-perpetuating vicious cycle [13]. The initial immune response in AD is significantly driven by epithelial-derived cytokines. Thymic stromal lymphopoietin (TSLP) acts as a master switch, released by keratinocytes in response to barrier damage or environmental insults [14]. TSLP promotes the maturation of dendritic cells that prime naive T cells to differentiate into a Th2 phenotype, thereby promoting the generation of canonical Th2-type cytokines including interleukin-13 (IL-13) [15]. As a downstream mediator, IL-13 directly represses the expression of the pivotal skin barrier protein loricrin, which further aggravates the disruption of epidermal barrier integrity [15]. Beyond the adaptive Th2 response, the innate immune system contributes to inflammation and pruritus. Pro-inflammatory cytokines like interleukin-1β (IL-1β), interleukin-6 (IL-6), and tumor necrosis factor-alpha (TNF-α) are elevated in AD lesions and are known to amplify local inflammation, recruit immune cells, and contribute to disease chronicity [16]. Furthermore, the histamine H4 receptor (HRH4) has emerged as a key mediator of pruritus and Th2 inflammation. Histamine plays roles in the induction of allergic inflammation by activating eosinophils, mast cells, basophils, and Th2 cells via HRH4, which is expressed on sensory neurons. A decrease in scratching behaviors was observed in HRH4-deficient mice and mice treated with a HRH4 antagonist [17].

Licorice is derived from the dried roots and rhizomes of Glycyrrhiza uralensis, Glycyrrhiza inflata, or Glycyrrhiza glabra. It has been widely used in commercial and pharmaceutical manufacturing as an empirical drug and healthcare product additive [18]. It has been proven that liquiritin (LQ), the active ingredient of licorice, has antidepressant [19], anti-cancer [20], anti-oxidation [21], and other pharmacological properties. Xia and colleagues demonstrated that LQ can improve immune dysregulation by reducing pro-inflammatory cytokine expression, thereby alleviating colitis and depression in mice [22]. Several studies also showed that LQ can reduce the production of inflammatory cytokine in SD rats and enhance the synthesis of collagen within the skin, thereby reinforcing the structural integrity of the epidermal barrier [23,24,25].

Our group previously developed liquiritin-based topical formulations, including a cold paste for UVB-induced solar dermatitis [26] and a carbomer gel (CG) for glucocorticoid-induced skin inflammation [27]. Although these formulations exhibited anti-inflammatory and skin reparative effects, their therapeutic potential in AD has not been explored. Other licorice-derived formulations have also been reported [28,29]; however, most lack comprehensive evaluation of both Th2-driven inflammation and skin barrier proteins as well as direct comparison with standard clinical drugs.

In contrast to the above studies (Table 1), the present work features a simple and easily prepared liquiritin carbomer gel (LQ-CG), which is the first such formulation to systematically investigate the dual regulation of skin barrier proteins and immune factors in a 2,4-dinitrofluorobenzene (DNFB)-induced AD mouse model. This is a formulation advancement. This study aims to investigate this formulation’s mechanisms in repairing the skin barrier and regulating immune dysregulation of AD both in vitro and in vivo experiments.

## 2. Results and Discussion

### 2.1. Ameliorative Effects of LQ-CG on DNFB-Induced Clinical Manifestations in Mice

**Figure 1:**
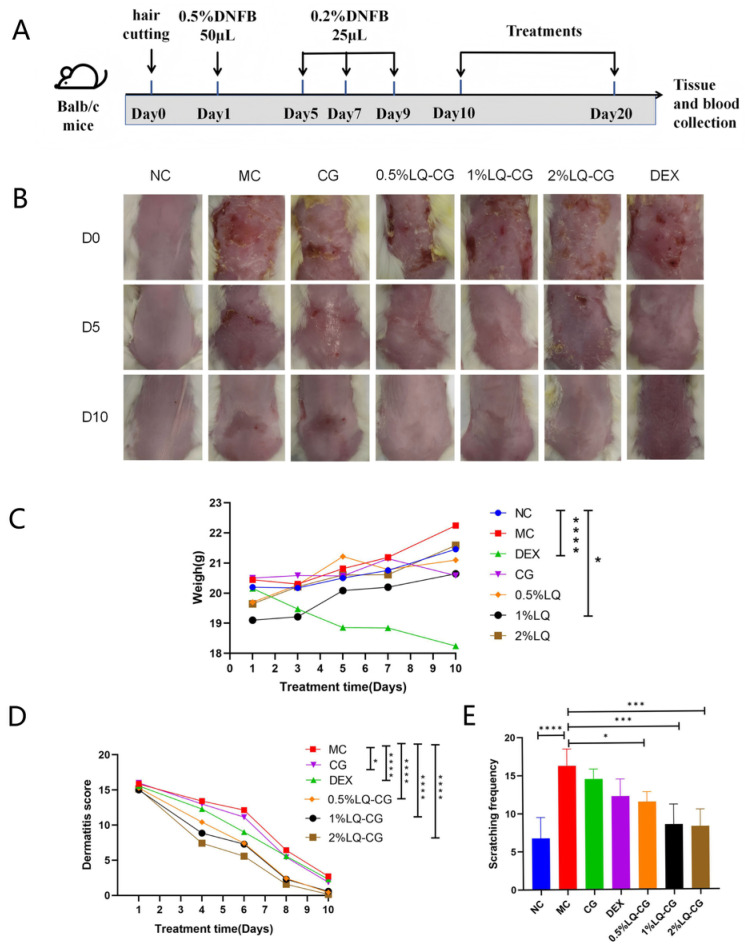
Effect of LQ-CG on DNFB-induced clinical features in mice. (**A**) Schematic diagram of the AD model establishment and treatment protocol. (**B**) Representative photographs of skin lesions. (**C**) Body weight changes. (**D**) Dermatitis severity score. (**E**) Scratching frequency within 20 min on day 10. NC (normal control), MC (model control), CG (vehicle control), DEX (dexamethasone, positive control group), and LQ-CG (liquiritin carbomer gel group). Data are expressed as the mean ± standard deviation (SD) (* *p* < 0.05, *** *p* < 0.001, **** *p* < 0.0001 *n* = 4).

By employing DNFB, we created an AD-like mouse model in Balb/c mice and later evaluated the effects of LQ-CG (Figure 1A). We observed that symptoms such as bleeding and edema, scratch erosion, dry desquamation, and skin covered with scattered scales were alleviated with both LQ-CG and DEX (dexamethasone) (Figure 1B). However, mice in the DEX group still showed dry desquamation and wrinkles. In addition, all LQ-CG treatment groups exhibited gradual weight gain throughout the treatment period, whereas the DEX group showed an average weight loss of 10% (Figure 1C). Furthermore, the application of LQ-CG decreased the dermatitis scores, and the 2% LQ-CG group showed the lowest dermatitis scores, indicating superior efficacy in repairing damaged skin (Figure 1D). Repeated DNFB administration triggered frequent scratching behaviors in mice, whereas treatment with LQ-CG or DEX significantly reduced the number of scratching episodes (Figure 1E).

### 2.2. LQ-CG Decreases Epidermal Thickness and Mast Cell Infiltration in Mice

**Figure 2:**
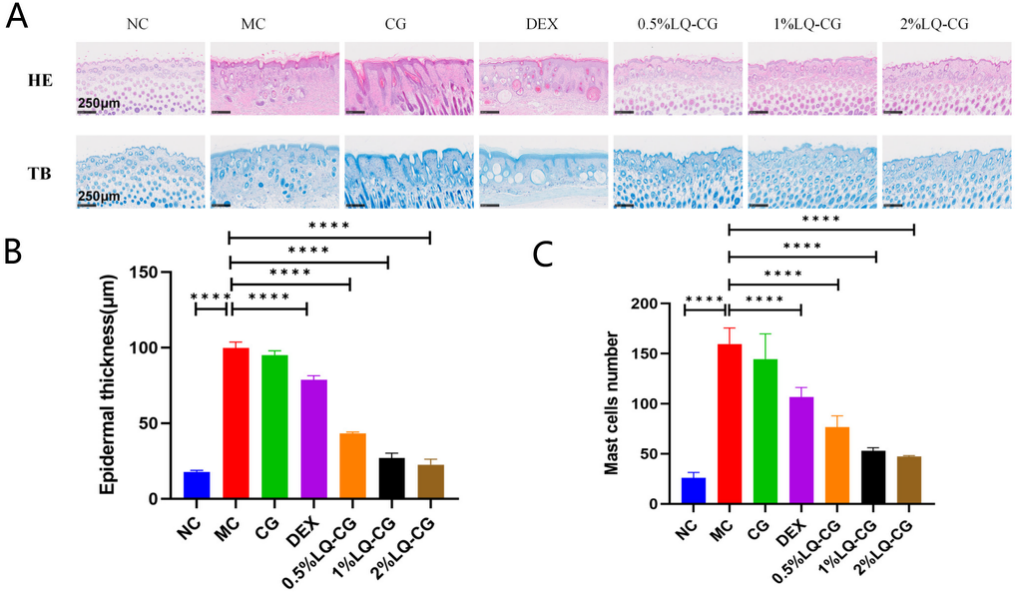
Effect of LQ-CG on skin histopathology in mice. (**A**) Representative micrographs of H&E staining (scale bar = 250 μm, ×10) and TB staining (scale bar = 250 μm, ×5). (**B**) Epidermal thickness. (**C**) Mast cell count. Data are expressed as the mean ± SD (**** *p* < 0.0001, *n* = 4).

Increased epidermal thickness and mast cell infiltration represent core pathological hallmarks of AD. By employing hematoxylin and eosin (H&E) and toluidine blue (TB) staining, we assessed the impact of LQ-CG on Balb/c mice treated with DNFB (Figure 2A). Compared with the NC (normal control) group, the skin thickness and mast cell infiltration of the MC (model control) group were significantly increased. Strikingly, LQ-CG treatment led to dose-dependent reductions in both epidermal thickness and mast cell infiltration. The 2% LQ-CG group achieved the optimal therapeutic effect and close to normal skin structure (Figure 2B,C).

### 2.3. LQ-CG Increases the Expression of Skin Barrier Proteins in Mice

**Figure 3:**
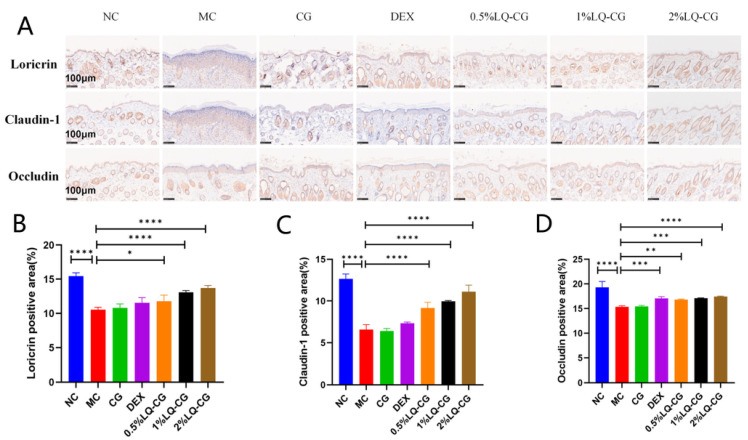
Effect of LQ-CG on skin barrier protein expression in AD-like skin lesions. (**A**) Representative immunohistochemical staining of loricrin, claudin-1, and occludin (scale bar = 100 μm, ×10) (**B**–**D**) Quantitative analysis of loricrin, claudin-1, and occludin protein expression levels. The data are shown as the mean ± SD (* *p* < 0.05, ** *p* < 0.01, *** *p* < 0.001, **** *p* < 0.0001, *n* = 4).

Immunohistochemistry (IHC) staining was used to analyze the expression of loricrin, occludin, and claudin-1 proteins, which are specifically related to AD and reflect skin barrier function and TJs in mouse skin (Figure 3A). Notably, LQ-CG demonstrated superior efficacy in increasing the expression levels of these proteins, particularly showing more pronounced and concentration-dependent recovery effects on loricrin and claudin-1. Specifically, the 2% LQ-CG group exhibited approximately double the claudin-1 expression level compared with the MC group. As key molecules in maintaining skin barrier function, the restoration of these proteins directly reflects the extent of barrier repair, further confirming LQ-CG’s potential in this context (Figure 3B–D).

### 2.4. LQ-CG Decreases Serum Inflammatory Cytokine Levels in Mice

**Figure 4:**
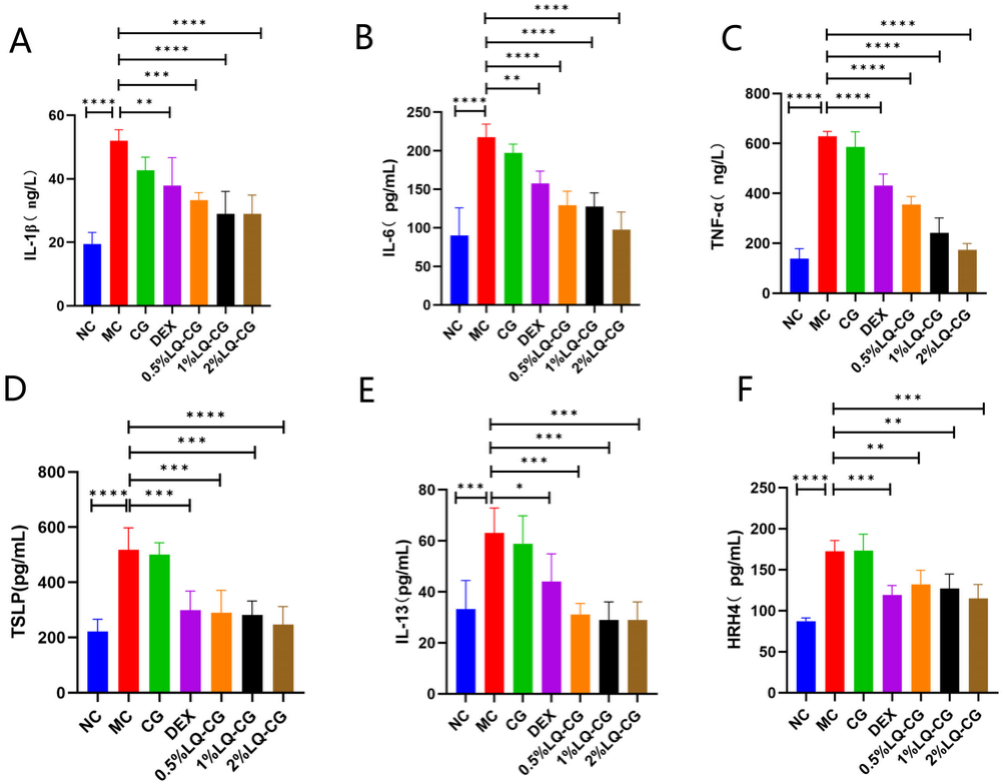
Effect of LQ-CG on serum inflammatory cytokines. (**A**–**F**) Serum levels of IL-1β, IL-6, TNF-α, TSLP, IL-13, and HRH4. Data are expressed as the mean ± SD (* *p* < 0.05, ** *p* < 0.01, *** *p* < 0.001, **** *p* < 0.0001, *n* = 4).

Serum levels of inflammatory cytokine serve as critical indicators for AD pathogenesis. Enzyme-linked immunosorbent assay (ELISA) was employed to study the influence of LQ-CG at different concentrations on inflammatory cytokine expression in an AD-like model mouse. In mice with DNFB exposed, we observed markedly elevated levels of pro-inflammatory cytokine including IL-1β, IL-6, and TNF-α (Figure 4A–C), along with Th2 immune cytokines TSLP, IL-13, and HRH4 (Figure 4D–F). Importantly, these cytokines were significantly suppressed in both the DEX and the LQ-CG treatment groups. In particular, the 2% LQ-CG group exhibited stronger inhibitory effects on most inflammatory cytokines, and all inflammatory cytokines recovered to levels close to the NC group, whereas the DEX group failed to achieve this. These findings indicate that LQ-CG attenuates AD-like symptoms by inhibiting the production of inflammatory cytokines.

### 2.5. LQ Suppresses Production of Inflammatory Cytokines In Vitro

**Figure 5:**
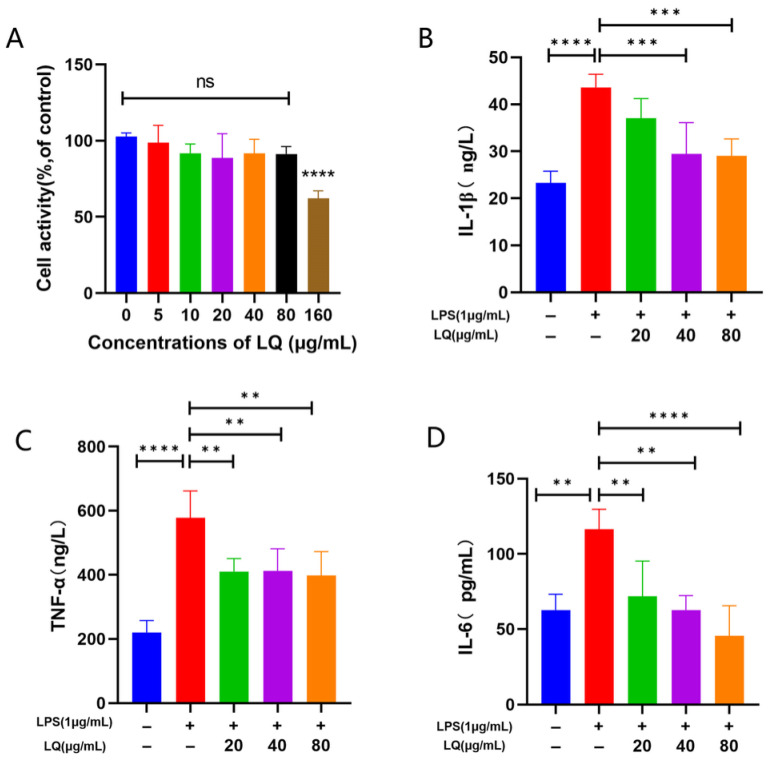
Effect of LQ on RAW264.7 cell viability and LPS-induced inflammatory cytokine secretion. (**A**) Cell viability of RAW264.7 cells. (**B**–**D**) Levels of IL-1β, TNF-α, and IL-6 expression. Data are presented as the mean ± SD (** *p* < 0.01, *** *p* < 0.001, **** *p* < 0.0001, *n* = 4).

In vitro experiments were performed to confirm the anti-inflammatory properties of LQ. Initially, the cytotoxicity of LQ in RAW264.7 cells was determined using the CCK-8 assay. Cell viability was significantly decreased at 160 μg/mL of LQ (Figure 5A), indicating that this concentration had toxic effects on the cells. Therefore, 80 μg/mL of LQ was the highest concentration used for subsequent experiments. Next, an ELISA was performed to determine the effects of different concentrations of LQ on the expression of inflammatory cytokines in RAW264.7 cells induced by lipopolysaccharides (LPSs). It was demonstrated that LPSs significantly increased the expression levels of inflammatory factors IL-1β, TNF-α, and IL-6 in RAW264.7 cells, while under 20, 40, and 80 μg/mL of LQ, these factors decreased significantly in a dose-dependent manner (Figure 5B–D). These results demonstrate that LQ exerted a significant anti-inflammatory effect in vitro.

### 2.6. Photographs of Spleens and Organ Indices of Mice

**Figure 6:**
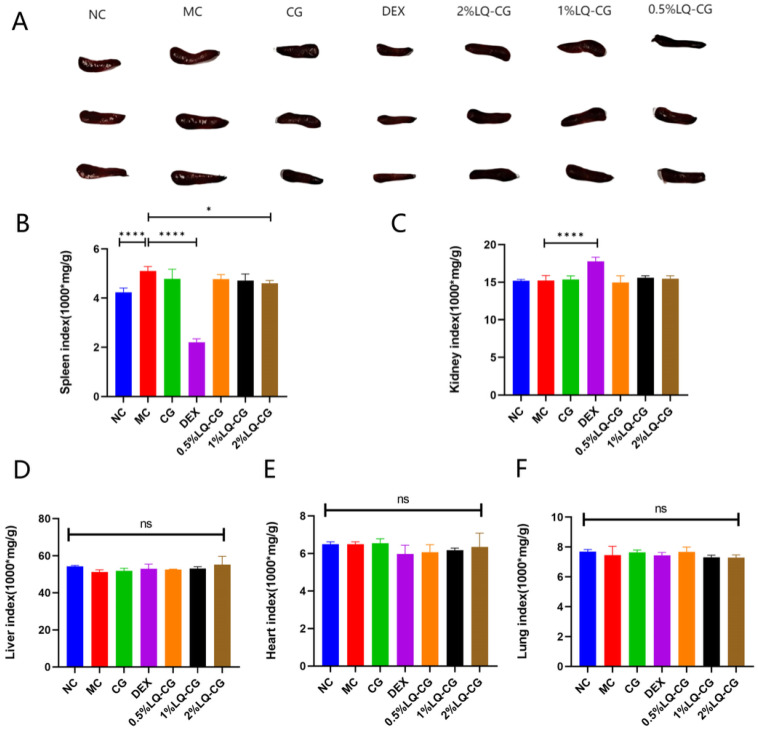
Spleen morphology and organ indices after 10-day treatment. (**A**) Representative photographs of spleen. (**B**–**F**) The spleen, kidney, liver, heart, and lung indices of mice in each group were measured. Data are expressed as the mean ± SD. “ns” denotes no discernible change (* *p* < 0.05, **** *p* < 0.0001, *n* = 4).

The spleen can be used as an indicator to measure the toxicity of drugs and their effects on the immune function of mice. Our results demonstrate that the spleen index was significantly elevated in the MC group compared with the NC group, whereas treatment with DEX markedly reversed this increase, suggesting abnormal immune function in AD-like mice and that DEX might inhibit immune response (Figure 6A,B). The organ indices in the LQ-CG groups were similar to that in the NC group. In addition, the kidney index of the DEX group was significantly increased compared with the MC group (Figure 6C), and there were no significant differences in the liver, heart, and lung indices (Figure 6D–F).

### 2.7. Discussion

AD is an allergic disease typically presenting with skin keratinization, pruritus, and impaired sleep quality, and is linked to other atopic diseases, such as asthma and allergic rhinitis [30]. Topical corticosteroids (TCSs) are used as the first-line treatment for AD, but patients are intolerant and fearful of TCSs due to their potential side effects [31]. The limitations of TCSs make it important to find a safe and effective natural treatment alternative to avoid the risk of long-term medication use. In contrast to phytochemical formulations that incorporate multiple active ingredients or complex excipients, the LQ-CG developed in this study consists solely of three components—liquiritin, carbomer, and triethanolamine—yet exerts significant anti-inflammatory effects and enhances skin barrier protein expression, with no notable alterations in body weight or major organ indices. In this study, in vitro and in vivo experiments showed that LQ-CG, a new type of Chinese herbal medicine-based external gel, had distinct advantages in AD treatment.

Initially, we established an AD-like mouse model using Balb/c mice and preliminarily confirmed that LQ-CG had a therapeutic effect on AD-like mice by observing skin changes and dermatitis scores. DEX also demonstrated significant efficacy in promoting skin healing in mice. However, during concurrent treatment, the DEX group exhibited skin wrinkles, dryness, and scaling, which may be attributed to DEX’s side effects [32]. Furthermore, the staining with H&E and TB of dorsal skin tissue in mouse models demonstrated that although both DEX and LQ-CG effectively improved skin thickness and mast cell infiltration, LQ-CG exhibited a more pronounced inhibitory effect, with both parameters reduced by approximately 50%, indicating stronger therapeutic potential. Notably, natural products or phytochemical-based formulations for AD have been used to target skin thickening and mast cell activation in many studies. Kim demonstrated that Pulsatilla koreana Nakai Extract alleviates AD-like symptoms in mice by reducing skin thickness and mast cell infiltration [33]. Similarly, Osthole decreased skin thickness in AD mice [34]. Wu reported that a hollow manganese dioxide-chitosan hydrogel exerted inflammation suppression, which was achieved by inhibiting epidermal thickening and reducing mast cell infiltration [35]. Consistent with these studies, LQ-CG exerted therapeutic effects by reducing epidermal thickness and mast cell infiltration. In our study, we mixed LQ with CG (carbomer gel), which may represent a more convenient and practical topical form for further application.

The pathogenesis of AD is complex and widely believed to involve both a compromised skin barrier and immune system imbalance. The skin serves as the primary barrier against the external environment of the body, exerting essential protective and homeostatic functions. The structure of the skin is divided into three layers from the outermost to the innermost region, namely the epidermis, dermis, and subcutaneous tissue, which collectively form the body’s initial barrier against environmental influences from outside [36]. The stratum corneum, which forms the outermost barrier of the skin, contributes to over 90% of the skin’s barrier function [37]. During the differentiation of epidermal keratinocytes from the basal layer to the stratum corneum, the synthesis of loricrin is a critical step. In the granular layer, cells initiate substantial synthesis of loricrin. As these cells differentiate into the outermost corneocytes, loricrin crosslinks with other proteins such as K1 and K10 at the cell periphery to form the keratinized envelope. Together with corneocytes and intercellular lipids, this envelope constitutes the “brick wall structure”, which protects against external stimuli and prevents water loss [38]. Skin barrier dysfunction is essential for the pathogenesis of AD. Studies have shown that reduced loricrin expression in the lesional skin of AD patients increases transepidermal water loss, thereby enhancing susceptibility to allergens [39]. Huang reported that Artemisia annua L. essential oil reversed the reduction in loricrin in DNCB-induced AD-like symptoms in mice [16]. In this study, LQ-CG treatment also significantly increased loricrin expression in mouse skin. In contrast to previous studies that only assessed loricrin, we further examined the expression of claudin-1 and occludin, which are essential TJs in the skin barrier.

TJs in the granular layer serve as complementary components of the skin barrier, restricting the penetration of allergens and pathogens through transmembrane proteins such as occludin and claudin-1 [40]. Within keratinocytes, TJs connect adjacent epidermal cells to form a permeability barrier [41]. Together, these mechanisms constitute the second physical barrier in the epidermis. Epidermal barrier impairment is a hallmark of AD. Downregulation of structural proteins, cornified envelope components, and TJ proteins disrupts the stratum corneum architecture. Such damage further compromises keratinocyte differentiation and physical defense mechanisms, thereby increasing skin susceptibility to allergens and microorganisms [36]. Several natural agents have been investigated for their ability to restore barrier function by enhancing claudin-1 and occludin expression. For instance, a tri-compound formula comprising ginsenoside Rg1, tetrandrine, and icariin has been shown to restore TJ barrier integrity by promoting the expression of claudin-1 and ZO-1 in the skin [42]. Likewise, Lithospermum erythrorhizon can ameliorate AD by restoring skin barrier function through increased expression of loricrin and occludin [40]. In our study, LQ-CG treatment significantly upregulated loricrin, occludin, and claudin-1 in mouse skin, which is similar to previous findings. Previous studies have primarily focused on only one or two of these proteins, providing a limited assessment of skin barrier recovery. In contrast, our study further examined Th2 immune response-related factors, including TSLP and IL-13, providing a more comprehensive correlation between immune modulation and barrier function and the protective effects of LQ-CG.

Another significant pathological mechanism in AD is immune dysregulation. Recent studies indicate that skin barrier dysfunction, immune abnormalities, and pruritus have reciprocal interactions among them [41]. When the skin barrier is impaired, allergens penetrate the epidermis and stimulate keratinocytes to release TSLP, which encourages the development of naive T cells into Th2 cells, and subsequently producing cytokines including IL-13 [15]. A previous study indicated that a hyaluronic acid hydrogel containing resveratrol-loaded chitosan nanoparticles can alleviate AD symptoms by inhibiting the release of TSLP and IL-13 [43]. In addition, TSLP has been shown to activate sensory neurons, leading to increased neuronal excitability and itch sensation, whereas IL-13 exacerbates pruritus both by directly affecting skin barrier proteins and by recruiting immune cells [44]. HRH4 plays a key role in the regulation of inflammatory pathways. Research has demonstrated that histamine binding to HRH4 on Th2 cells increases the production of IL-13, and the enhanced expression of HRH4 signifies its crucial function in inducing allergic inflammation [36]. Histamine induces pruritus by activating HRH4 on sensory nerves [45]. Scratching aggravates cutaneous inflammation and immune responses; mechanical damage to keratinocytes triggers pro-inflammatory reactions and facilitates immune cell infiltration, thereby establishing a vicious “itch–scratch–itch” cycle. In a prospective clinical study, inhibition of IL-13 demonstrated the potential to reduce epidermal hyperplasia and suppress pro-inflammatory cytokine production in AD patients [46]. Another study revealed that inhibiting HRH4 expression may alleviate the progression of AD [47]. These studies suggest that targeting IL-13, TSLP, and HRH4 may represent a potential strategy for AD management. In this study, LQ-CG significantly reduced the expression of these factors, suggesting a potential association between LQ-CG treatment and suppression of Th2 inflammation. However, as the present study was designed primarily to evaluate efficacy, precise molecular mechanisms remain unknown and warrant further investigation.

To further evaluate the anti-inflammatory potential of LQ-CG, we conducted both in vivo and in vitro experiments. Accumulating evidence has established that inhibiting the production of IL-6, TNF-α, and IL-1β can be a key therapeutic strategy for AD. For instance, the active fractions of Wuwei Xiaodu decoction can reduce inflammatory cytokines in LPS-induced RAW264.7 cells [48]. Specific probiotics alleviate AD-like symptoms in mice by concurrently reducing epidermal hyperkeratosis, mast cell infiltration, and the expression of TNF-α, IL-6, and IL-1β [49]. Similarly, Kim demonstrated that pharmacological inhibition of these cytokines effectively ameliorates AD pathology [29]. More recently, Zhang reported that a Vaccinium vitis-idaea liposomal gel ameliorated AD symptoms by reducing the expression levels of TNF-α [50]. Consistent with these reports, our in vitro results confirm that LQ-CG significantly suppresses the expression of the above inflammatory cytokines. Importantly, this anti-inflammatory effect was further validated in vivo, as evidenced by reduced serum levels of the same cytokines. Notably, compared to formulations such as the liposomal gel reported by Zhan [50], which targeted a broader cytokine spectrum, LQ-CG appeared to specifically and effectively modulate the classical TNF-α, IL-6, and IL-1β axis, suggesting a potentially beneficial mechanism needing further investigation.

Chronic inflammation, often associated with excessive immune activation, can lead to changes in immune organs such as the spleen. Studies have demonstrated a significant elevation in the splenic index in AD-like mice, suggesting potential abnormal activation of the immune response [51]. In the present study, the spleen index was significantly reduced in the DEX group compared with the MC group (*p* < 0.001), consistent with the immunosuppressive effect of TCS. Moreover, the kidney index in DEX-treated mice was elevated, suggesting a potential for renal impairment [52], though further investigation is needed. Together with the observed changes in skin texture and thinning, these alterations are consistent with the known side effects of TCS. In contrast, no significant changes in organ indices were observed in the LQ-CG-treated groups (*p* > 0.05 for all comparisons), suggesting no obvious immunosuppressive effect, supporting its safety profile. A previous study by Tan demonstrated that bacterial cellulose-based glycyrrhizic acid gel significantly alleviated abnormal splenic hyperplasia, suggesting that this hydrogel may improve AD-like symptoms by inhibiting spleen proliferation [53]. Similarly, LQ-CG treatment also reversed AD-induced splenomegaly with no significant changes in organ indices observed. In summary, LQ-CG presents a promising therapeutic strategy for AD that can mitigate skin lesions without the typical side effects of TCS therapy.

Notably, LQ-CG treatment exhibited superior efficacy in alleviating AD-like symptoms compared with the DEX group. LQ-CG more effectively reduced skin thickness and mast cell infiltration, upregulated skin barrier protein expression, and decreased inflammatory cytokine production, with no significant differences in organ indices. We observed that higher concentrations of LQ-CG (2%) consistently showed greater efficacy compared with lower concentrations (vs. 0.5% and 1%, *p* < 0.05), suggesting a concentration-dependent effect. In comparison to long-term TCS use, these findings support its potential as a natural alternative to TCS. However, the potential molecular mechanisms require further clarification. This exploratory study had a small sample size (*n* = 4 per group); larger-scale studies are needed to confirm these findings. The DNFB-induced mouse model used in this study is a well-established and widely accepted model for AD research as it recapitulates key features of human AD including epidermal thickening, immune cell infiltration, and Th2-skewed immune responses [54]. However, it cannot fully mimic the genetic complexity and chronicity of human AD. Furthermore, our in vitro anti-inflammatory evaluation was performed using LPS-stimulated RAW264.7 cell macrophages, which mainly reflect innate immune responses rather than the Th2 immune microenvironment of AD. The study also lacked keratinocyte-based mechanical assays and in vitro mechanistic tests on Th2 cytokines. Therefore, future research using more models related to AD and samples is needed to confirm the translational potential of these findings.

## 3. Conclusions

This study demonstrated that LQ-CG effectively alleviated DNFB-induced AD-like skin lesions in mice, inhibits pro-inflammatory cytokine production, and enhances skin barrier protein expression. Despite the limitations discussed above, these findings suggest that LQ-CG may mitigate AD-like symptoms by suppressing inflammatory response and restoring skin barrier function. It provides a potential application strategy for the clinical treatment of AD. Therefore, these results indicate that LQ-CG may serve as a potential therapeutic approach for AD and warrants further investigation for future clinical application. However, further research is required to clarify its potential mechanisms.

## 4. Materials and Methods

### 4.1. Preparation of LQ-CG

We employed the method previously established by our research team to prepare the LQ-CG [26,27]. Briefly, we combined 1 g of carbomer 940 powder (Meilunbio, Dalian, China) with 100 mL of ultrapure water and stirred the mixture for 5 min to form 1% CG. Then, we slowly added triethanolamine (Macklin, Shanghai, China) until the pH was adjusted to 7.0, measured using precision pH paper. Subsequently, different weights of LQ (Chengdu Nakeli Biotechnology Co., Ltd., Chengdu, China) were dissolved in sodium carboxymethyl cellulose (CMC-Na, Macklin, Shanghai, China) solution and incorporated into 1% CG to achieve final concentrations of 0.5%, 1%, and 2% (*w*/*v*). The mixture was stirred continuously at 25 °C until a homogeneous and stable gel was formed. Our research team previously demonstrated that LQ-CG exhibited a uniform porous structure, with a cumulative drug release rate of up to 55.34% within 4 h, and maintained stability in temperatures ranging from −20 °C to 60 °C [26,55].

### 4.2. Modeling and Treatment of AD-like Mouse

All experimental animal procedures followed the Laboratory Animal Care and Use Guidelines of Guangdong Pharmaceutical University. The research was approved by the Animal Ethics Committee (approval code: gdpulacspf2022738 approval date: 3 January 2025). A total of 28 (*n* = 4 per group) SPF (specific pathogen-free) female Balb/c mice weighing 16–18 g, aged 6–8 weeks, were purchased from the Guangdong Provincial Experimental Animal Center. Sample size was determined based on previous studies [56,57,58] with similar experimental designs. Animals were housed in the SPF experimental animal facility under the following controlled conditions: temperature of 22–24 °C, relative humidity of 40–60%, and a 12-h light/dark cycle. We frequently changed the bedding and provided adequate feed and clean drinking water. After one week of adaptive feeding, an area of approximately 2 cm × 2 cm on the dorsal skin was shaved the day before modeling. With the exception of the normal NC group, the mice in all other groups were topically administered 50 μL of 0.5% DNFB (Macklin, Shanghai, China) on day 1, and 25 μL of 0.2% DNFB was applied for sensitization and challenge on days 5, 7, and 9. The mice were observed every day, and erythema, edema, bleeding, epidermal shedding, scabbing, and desquamation indicated that the model was successfully constructed. The modeling mice were equally divided into six groups (*n* = 4 per group) using a random number table: the MC group, CG group, 0.5%LQ-CG group, 1%LQ-CG group, 2%LQ-CG group, and DEX group (dexamethasone, China Resources Sanjiu Pharmaceutical Co., Ltd., Shenzhen, China). The NC and MC groups were not treated, while the other groups were treated with corresponding reagents on the skin, applied once a day for 10 days. To minimize bias, the investigator responsible for treatment administration, clinical scoring, and data analysis was blinded to group allocation. Our primary outcome measures included dermatitis severity score and scratching frequency, and measured as follows: the skin of the mice was photographed at the same height, and body weight was measured every day. The dermatitis score included four aspects: erythema/hemorrhage, excoriation/erosion, edema/exudation, and dryness/scaling/lichenification. A score of 0 to 4 was given according to the affected area (absent = 0, <10% = 1, 10–40% = 2, 40–75% = 3, and >75% = 4), and the total score (0–16) was recorded. At the end of the treatment, the mice were placed in transparent boxes, and the scratching frequency was recorded for 20 min using a high-definition camera (Canon Inc., Tokyo, Japan). Finally, the mice were sacrificed after anesthesia with isoflurane. Biopsies of skin tissue were used for pathological examination, and organ tissue was used to calculate the organ index.

### 4.3. ELISA

The blood gained from the eyeball of a mouse was placed in a 2 mL centrifuge tube, and centrifuged for 20 min at 2000 revolutions per minute after a 2-h pause to obtain the serum. The serum levels of TNF-α, IL-1β, IL-6, TSLP, IL-13, and HRH4 were measured using ELISA kits (Jiangsu Meimian Industrial Co., Ltd., Yancheng, China) following the manufacturer’s protocols.

### 4.4. Skin Histopathological Evaluation and Histological Analysis

Dorsal skin specimens from each mouse were collected and fixed in 4% paraformaldehyde (Biosharp, Bengbu, China) for 24 h. The tissue samples were then dehydrated, embedded in paraffin, and cut into 4 μm sections using a microtome (Wuhan Junjie Electronics Co., Ltd.,Wuhan, China). Subsequently, the sections were subjected to staining with H&E and TB.

Additionally, IHC was employed to examine the protein expression levels of claudin-1, loricrin, and occludin in skin tissues from mice. The experimental procedures were conducted as follows: sections were first deparaffinized. The samples were then incubated with the primary antibody at 4 °C overnight. Subsequently, the samples were stained using 3,3′-diaminobenzidine tetrahydrochloride solution and goat anti-rabbit IgG-HRP conjugate and counterstained with hematoxylin.

Primary antibodies included claudin-1 (ab15098, Abcam, Cambridge, UK), loricrin (55439-1-AP), and occludin (27260-1-AP) from Proteintech (Chicago, IL, USA). Images were captured using a microscope and slide scanner, and quantitative analysis was performed with ImageJ software (version 1.54p).

### 4.5. Culture of RAW264.7 Cells

The RAW264.7 cell line was purchased from Procell Life Science and Technology Co., Ltd. (Wuhan, China). Cells were maintained in Dulbecco’s modified Eagle’s medium (DMEM; GIBCO, Grand Island, NE, USA) supplemented with 10% fetal bovine serum (FBS; BIOIND, Kibbutz Beit Haemek, Israel) and 1% penicillin-streptomycin solution (100 U/mL penicillin and 100 U/mL streptomycin; GIBCO). All cells were incubated at 37 °C in a humidified atmosphere containing 5% CO_2_.

### 4.6. Cell Cytotoxicity Assay and ELISA

CCK-8 (Meilun Biotechnology, Dalian, China) was used to detect the viability of RAW264.7 cells. The cells were planted into 96-well plates at a density of 10,000 cells per well, with 100 μL of solution added to each well. Following 12 h of incubation, the cells were treated with various concentrations of LQ. After 24 h of incubation, we added 10 μL of CCK-8 solution to each well. Absorbance at 450 nm was measured using a microplate reader (Biotek, Winooski, VT, USA) after 1 h of incubation.

ELISA kits (Jiangsu Meimian Industrial Co., Ltd., Yancheng, China) were used to detect the levels of TNF-α, IL-1β, and IL-6. RAW264.7 cells were first incubated with LQ for 4 h, stimulated with LPS for an additional 12 h, and then the supernatants were collected and centrifuged at 4 °C for 10 min. All assays were performed following the manufacturer’s protocols.

### 4.7. Statistical Methods

All data are expressed as the mean ± SD. The Shapiro–Wilk test was used to confirm normality before analysis. GraphPad Prism (version 9.5.1, GraphPad Software, Inc., San Diego, CA, USA) was used to analyze the data, and a one-way ANOVA followed by Dunnett’s post hoc test was used to compare each group with the model group. A value of *p* < 0.05 was defined as statistically significant.

## Figures and Tables

**Table 1:** Comparison of representative topical formulations based on licorice-derived compounds for inflammatory skin diseases.

Ref.	Active Agent	Dosage Form	Disease Model	Key Findings	Limitations
[26]	Liquiritin	Carbomer gel cold paste	UVB-induced solar dermatitis	Anti-inflammatory, wound healing	Not applicable to AD, different dosage form
[27]	Liquiritin	Carbomer gel	Glucocorticoid-induced skin inflammation	Barrier restoration, reduced inflammation	Not evaluated in AD, no Th2 cytokine analysis
[28]	Licorice extract	Self-assembled nanoparticles	2,4-Dinitrochlorobenzene (DNCB)-induced atopic dermatitis	Reduced histopathology and cytokines	Multi-component extract, complex preparation, no single active comparison
[29]	Glycyrrhizic acid, liquiritin, oxymatrine	Microemulsion gel	DNCB-induced chronic eczema	Reduced ear swelling and inflammation	Multi-component, disease model not AD, no DEX comparison
This work	Liquiritin	Carbomer gel	DNFB-induced atopic dermatitis	Upregulated barrier proteins, suppressed Th2 cytokines, comparable efficacy to DEX, and no organ index changes	Small sample size, and no mechanistic pathways

## Data Availability

The datasets are not publicly available due to ongoing intellectual property protection. However, they are available from the corresponding author upon reasonable request.

## Abbreviations

The following abbreviations are used in this manuscript:

AD	Atopic dermatitis
LQ	Liquiritin
LQ-CG	Liquiritin carbomer gel
DNFB	2,4-Dinitrofluorobenzene
LPS	Lipopolysaccharide
TJs	Tight junctions
Th2	T-helper 2
TSLP	Thymic stromal lymphopoietin
IL-13	Interleukin-13
IL-1β	Interleukin-1β
IL-6	Interleukin-6
TNF-α	Tumor necrosis factor-alpha
HRH4	Histamine H4 receptor
CG	Carbomer gel
DNCB	2,4-Dinitrochlorobenzene
DEX	Dexamethasone
NC	Normal control
MC	Model control
SD	Standard deviation
H&E	Hematoxylin and eosin
TB	Toluidine blue
IHC	Immunohistochemistry
ELISA	Enzyme-linked immunosorbent assay
TCSs	Topical corticosteroids
SPF	Specific pathogen-free
